# A Lower False Positive Pulmonary Nodule Detection Approach for Early Lung Cancer Screening

**DOI:** 10.3390/diagnostics12112660

**Published:** 2022-11-01

**Authors:** Shaohua Zheng, Shaohua Kong, Zihan Huang, Lin Pan, Taidui Zeng, Bin Zheng, Mingjing Yang, Zheng Liu

**Affiliations:** 1 College of Physics and Information Engineering, Fuzhou University, Fuzhou 350108, China; 2 School of Future Technology, Harbin Institute of Technology, Harbin 150000, China; 3 Key Laboratory of Cardio-Thoracic Surgery (Fujian Medical University ), Fujian Province University, Fuzhou 350108, China; 4 School of Engineering, Faculty of Applied Science, University of British Columbia, Kelowna, BC V1V 1V7, Canada

**Keywords:** pulmonary nodule detection, false positive reduction, multi-scale object detection, convolutional neural network, computer-aided detection system

## Abstract

Pulmonary nodule detection with low-dose computed tomography (LDCT) is indispensable in early lung cancer screening. Although existing methods have achieved excellent detection sensitivity, nodule detection still faces challenges such as nodule size variation and uneven distribution, as well as excessive nodule-like false positive candidates in the detection results. We propose a novel two-stage nodule detection (TSND) method. In the first stage, a multi-scale feature detection network (MSFD-Net) is designed to generate nodule candidates. This includes a proposed feature extraction network to learn the multi-scale feature representation of candidates. In the second stage, a candidate scoring network (CS-Net) is built to estimate the score of candidate patches to realize false positive reduction (FPR). Finally, we develop an end-to-end nodule computer-aided detection (CAD) system based on the proposed TSND for LDCT scans. Experimental results on the LUNA16 dataset show that our proposed TSND obtained an excellent average sensitivity of 90.59% at seven predefined false positives (FPs) points: 0.125, 0.25, 0.5, 1, 2, 4, and 8 FPs per scan on the FROC curve introduced in LUNA16. Moreover, comparative experiments indicate that our CS-Net can effectively suppress false positives and improve the detection performance of TSND.

## 1. Introduction

**Figure 1:**
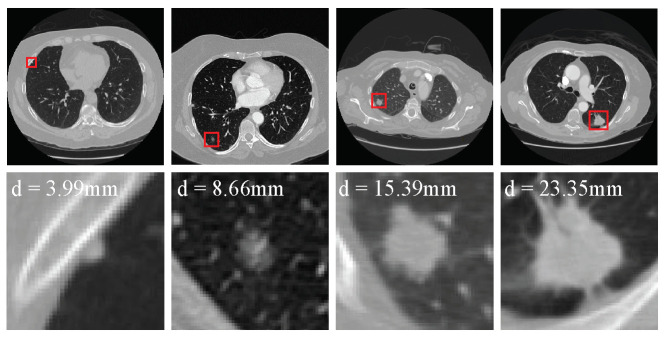
Examples of the nodules in the LUNA16 dataset. The first row shows the nodules (red boxes) from four raw LDCT scans. Meanwhile, each nodule is zoomed in and shown in the second row.

Lung cancer is among the most common malignancies, approximately 2.2 million new lung cancer cases and 1.8 million deaths were recorded worldwide in 2020 [1]. Making early inspection and diagnosis of lung cancer is significant for its treatment and prognosis [2,3]. However, the early symptoms of lung cancer are not obvious [4]; thus, causing patients easily miss an optimal treatment period. Pulmonary nodules are abnormal lesions, usually but not all spherical (Figure 1); and they are also signs of suspected lung cancer [5]. Pulmonary nodules are abnormal lesions with spherical (Figure 1). These are among the early clinical symptoms of lung cancer [5]. Early detection followed by timely diagnosis and treatment can effectively improve the five-year survival rate of lung cancer [6].

Low-dose computed tomography (LDCT) imaging is a common and effective tool for early lung cancer screening with less radiation dose than conventional CT because it has less radiation dose and faster scanning speed than conventional CT [7]. Considering the massive amounts of scans that need to be diagnosed, and each scan has hundreds of 2D slices, manual inspection is a time-consuming and tedious task for clinicians. Moreover, pulmonary nodules are easily confused with blood vessels, airway walls, and lung aberrations, due to the heterogeneity of nodules [8,9], such as variations in size and location, blurred outlines, and low contrast. In conclusion, manual pulmonary nodule screening is a laborious task, and prone to missed diagnosis, especially for small nodules [10]. Overall, it is difficult for clinicians to quickly and exactly recognize nodules [10].

Various computer-aided detection (CAD) systems have been designed to enable clinicians to quickly and accurately diagnose pulmonary nodules [11]. The CAD system first scans and interprets 3D LDCT scans, and then provides candidates of suspected pulmonary nodules [12]. Traditional CAD systems mainly have relied on feature engineering to design feature extractors to generate nodule candidates [13,14,15]. However, these systems often fail to achieve good generalization, because the low-level features extracted by them do not adapt well to various changes in lung nodules, such as size, shape, and density [16]. However, these systems are often not well-generalized, Because nodules are heterogeneous. With the rise of deep learning, convolutional neural networks (CNNs) have been designed and applied to natural image analysis tasks with great success. In the nodule detection, CNN-based methods [9,12,16,17,18,19,20,21,22,23,24] are much better than traditional methods and have high detection sensitivity. Because CNNs can continuously learn and optimize feature representations of nodules from LDCT scans to enhance their detection generalization.

There are several difficulties in constructing CNN-based methods to detect nodules in 3D LDCT scans. First, compared with object detection in 2D natural images the context information of nodules is complex because nodules are 3D objects, often produce adhesion with different tissues, and have a variety of attributes (e.g., density and edge) in 3D scans. Figure 1 shows some examples of the nodules in the lung analysis 2016 (LUNA16) challenge dataset [25,26]. Several papers propose one-stage end-to-end 3D CNN methods [9,12,27] to effectively detect nodules in the LDCT scans.

**Figure 2:**
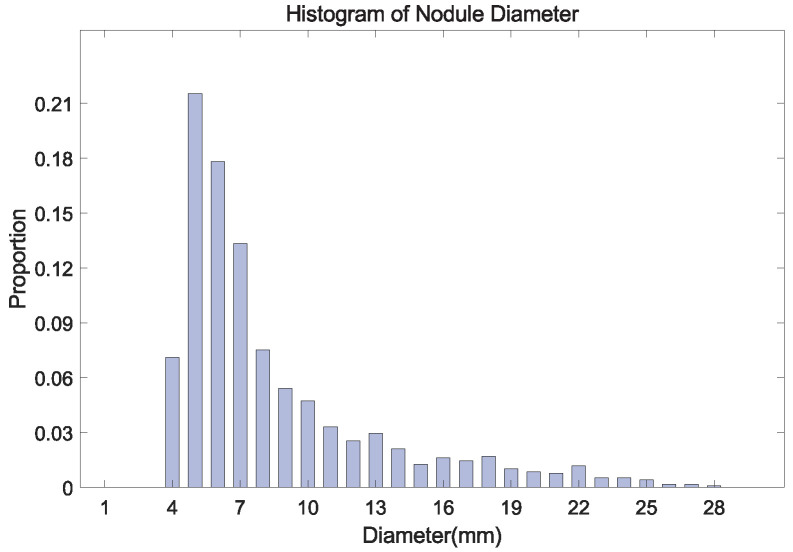
The histogram of nodule diameter (mm). The average diameter is 8.31 mm for all nodules, in the LUNA16 dataset.

Second, The size of nodules is unevenly distributed in the range of 3∼30 mm, where the number of small nodules is the largest (Figure 2). The number of labeled nodules is limited due to the difficulty of collecting and labeling nodule data being difficult [12]. These problems often result in the over-fitting of the model obtained in the training stage. Some studies proposed to embed attention mechanisms in the model [22,23,24], which helps the model to learn more effective features.

Third, many nodule-like tissues exist in the lung, which leads to many false positive candidates. This issue reduces the precision of nodule detection. Several two-stage nodule detection methods [16,18,21,28] are proposed to deal with this issue. In addition, some researchers adopt CNNs to build candidate classification networks [29,30,31,32,33] that effectively suppress false positives in nodule detection.

In this paper, we propose a novel two-stage nodule detection (TSND) method based on 3D CNN modules. It can automatically predict nodule candidates and suppress false positive candidates to precisely assist clinicians in early pulmonary nodule diagnosis using 3D LDCT scans. Our contributions are summarized as follows:

1. We propose an architecture called a multi-scale feature extractor (MSFE) to learn multi-scale feature representations of nodule candidates from training data. Based on MSFE, an anchor-box multi-scale feature detection network (MSFD-Net) is proposed to generate nodule candidates. It adapts well to detection difficulties caused by the heterogeneity of nodules through a multi-scale detection strategy.
2. We build a candidate scoring network (CS-Net) to evaluate the confidence score of candidates. In our TSND, we use the CS-Net to estimate the score of candidates provided by an MSFD-Net to realize the false positive reduction.
3. We develop an end-to-end nodule computer-aided detection (CAD) system to help with early nodule diagnosis. This integrates a preprocessing and TSND module. It can directly provide the detected nodule from raw LDCT scans.

We evaluate our methods on the LUNA16 dataset using 10-fold cross-validation. Experimental results indicate that our TSND obtains superior performance compared with existing state-of-the-art methods. Meanwhile, the CS-Net can effectively suppress false positive candidates to improve detection performance.

The remaining sections of this paper are constructed as follows. Section 2 describes the related works of nodule detection based on CNNs. Section 3 presents our adopted dataset and proposed solution, including the LUNA16 dataset, evaluation metrics, nodule CAD system, the architectures of networks, and the details of the training used. The experimental results and detailed analysis are introduced in Section 4 and Section 5. The conclusion is presented in Section 6.

## 2. Related Work

Early pulmonary nodule detection is an effective way to reduce the number of new cases and deaths related to lung cancer. Currently, the CNN-based model has become the mainstream detection method because it can mine the high-level features of the detected object from the data. Thus the model has better generalization than the traditional method [16]. Lung analysis 2016 challenge dataset [26], consisting of a number of labeled 3D LDCT scans, is used to develop various CNN-based computer-aid detection methods. Most of the CNN-based nodule CAD methods usually include two tasks: nodule candidate detection and false positive reduction [23]. Nodule candidate detection achieves the screening of suspected nodules to ensure the sensitivity of nodule detection, and false positive reduction achieves the elimination of non-nodules to improve the precision of nodule detection. Some related works on nodule detection are introduced below and are listed in Table 1.

Lung nodules are 3D objects in 3D scans. As shown by object detection methods in natural images [34,35,36,37]. Some researchers have used these methods to realize an efficient one-stage nodule detection [9,12,20,22,23,24,27,38] with excellent detection speed and sensitivity. Liao et al. [12] developed a 3D CNN-based nodule detection nodule method (N-Net), which first used a 3D U-Net as a backbone to extract the features of nodules and adopted a 3D RPN to predict nodule candidates. Based on an 3D ResNet-18, Li et al. [22] proposed an end-to-end 3D deep CNN with encoder-decoder structure (DeepSEED) to detect the nodule. They introduced squeeze-and-excitation attention [39] to the residual block. Thus, the network learns effective features and ultimately improves the detection performance of nodules. Luo et al. [23] proposed an anchor-free 3D sphere representation center-points matching network (SCPM-Net) for nodule detection by regarding a pulmonary nodule as a sphere. SCPM-Net predicts center point mapping, location offset, and the diameter of candidates in LDCT scans.

The one-stage method focuses on ensuring the sensitivity of nodules. However, the detected candidates contain a number of false positives. Some researchers have designed a false positive reduction module that is embedded into a one-stage detection network and proposed two-stage nodule detection methods [16,17,18,19,21,28]. Tang et al. [21] proposed an end-to-end multi-task network framework named as NoduleNet. NoduleNet first initially generates candidates by a U-Net [40]. Then, two-branched sub-networks share the features extracted by the U-Net, which achieve false positive reduction and candidate segmentation, respectively. Mei et al. [16]. designed a CNN network called as Slice-Aware Network (SA-Net) to achieve two-stage pulmonary nodule detection. In SA-Net, nodule candidates are first generated by a feature extractor and RPN with high confidence. Then, a false positive reduction module is used to re-identify candidates and correct the position of candidates using the multi-scale features from the feature extractor. Ding et al. [18] first designed a nodule detection approach based on Faster Region-based CNN (FRCN). Then, they implemented a 3D CNN to remove false positive candidates generated by FRCN.

In summary, the one-stage method has a simple structure and is easy to implement. In terms of detection performance, the one-stage method has high detection sensitivity and fast inference speed. However, the generated candidates contain many false positives. While the two-stage approach has a strategy to reduce false positives and can provide physicians with more accurate nodule candidates, it is more complicated in structure and implementation.

## 3. Materials and Methods

### 3.1. Materials

**LUNA16 Dataset.** In this paper, the publicly available LUNA16 dataset is used to fit proposed networks and evaluate the performances of proposed methods for nodule detection. In LUNA16, a total of 888 LDCT scans with a spacing of less than 3 mm are included and divided into 10 subsets by the official for the 10-fold cross-validation [25,26]. In addition, there are two labeled datasets for nodule detection provided, as follows.

First, a detection dataset that contains 1186 pulmonary nodule labels (coordinates and diameter) is provided. It aims to build complete nodule detection methods. In addition, LUNA16 also furnished an excluded nodule dataset, which includes multiple nodules, pulmonary inflammation, and suspected nodules.

Second, LUNA16 offers a large-scale dataset of nodule candidates CandidatesV2 (CandisV2). Its purpose is to establish a false positive reduction method after nodule detection. CandisV2 includes a total of 754,975 nodule candidate labels (coordinates and class), uniformly distributed in the 888 LDCT scans, where the number of positive and negative samples is 1557 and 753,418, respectively.

**Evaluation Metrics.** In the LUNA16 Challenge, the performance of nodule CAD systems is evaluated by the Free-Response Receiver Operating Characteristic (FROC) curve calculated, of which the ordinate is sensitivity, and there is an average number of false positives per scan (FPs/Scan) for the ordinate [26]. The FROC curve reflects the CAD system’s ability to detect the sensitivity of nodules and restrict false positives.

In addition, the Competition Performance Metric (CPM) value is defined as the average sensitivity at seven FPs points, i.e., 0.125, 0.25, 0.5, 1, 2, 4, and 8 FPs per scan on the FROC curve. This value is adopted to measure the final detection performance of the CAD system. Obviously, an outstanding detection system will gain an average sensitivity close to 100%, and the lowest sensitivity is 0% [25]. In this work, we similarly adopt FROC values at seven FPs/Scan points and the CPM value described above to evaluate our proposed solution. We compare our results with those of state-of-the-art detection methods.

### 3.2. The Proposed Nodule Computer-Aided Detection System

**Figure 3:**
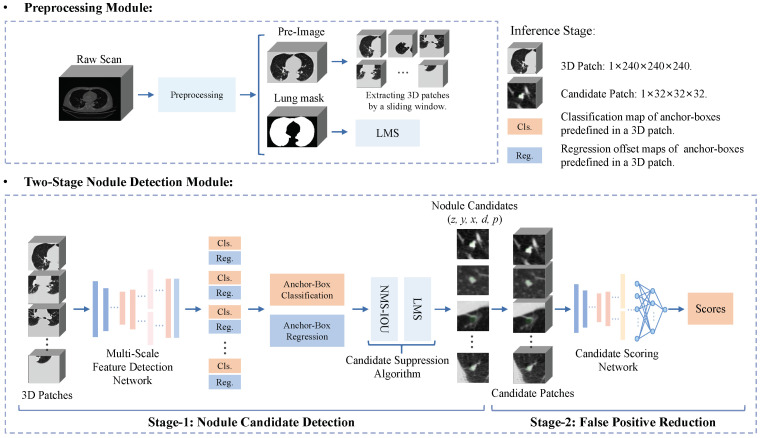
Overall flowchart of the proposed end-to-end nodule CAD system. It includes a preprocessing module to identify a region of interest in a raw LDCT scan, and a two-stage nodule detection module to generate nodule candidates and reduce false positives.

**Figure 4:**
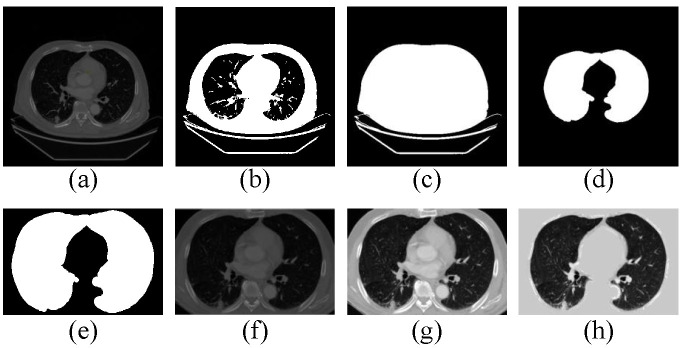
The procedure of preprocessing for raw LDCT images.

Figure 3 shows the overall flowchart of our proposed CAD system that includes a preprocessing module and two-stage nodule detection (TSND) module. First, the preprocessing module identifies a region of pulmonary parenchyma from a fed raw LDCT scan, and a lung mask and pre-image are obtained by the remaining procedure. Details are shown in Section 3.2.1. Second, the TSND precisely finds out nodule candidates from the pre-image with high sensitivity. Details are shown in Section 3.2.2. Finally, all the detected results are provided to clinicians to provide reference information for the diagnosis of pulmonary nodules. In addition, Algorithm 1 detailed illustrates the inference procedure of our proposed CAD system in a detailed manner for elucidation.
**Algorithm 1** Pseudo code of the proposed nodule CAD system.**Input:** 
A raw LDCT scan, $I_{raw}$; The procedure of preprocessing, Procer; 3D patch extractor, Tailor; Nodule candidate detection, Detector; False positive reduction, Estimator.**Output:** 
a list of nodules detected from the $I_{raw}$, $List_{n}$1:**Tokens & Initialization:**A 3D Patch $I_{patch}$ with shape of $Shape_{patch}:\left[1,240,240,240\right]$;A candidate patch $I_{candi}$ with shape of $Shape_{candi}:\left[1,32,32,32\right]$;A starting coordinate of a $I_{patch}$ in $I_{pre}$;A set of predefined anchor-boxes, $Set_{ABox}$;A candidate threshold of Detector, $C_{th}:0.75$;A candidate score of Estimator, $C_{s}:0.05$;A candidate set, $Set_{candi}$;2:/*Preprocessing Module*/3:Procer: Pulmonary parenchyma mask is extracted from the $I_{raw}$, as shown in Figure 4a–d;4:Procer: The $I_{pre}$ and $I_{mask}$ are obtained, as shown in Figure 4e–h;5:/*Two-Stage Nodule Detection Module*/6:Generates $Coord_{patch}$ of each 3D patch in $I_{pre}$ and stores them in a coordinate list $List_{coord}$;7:**for** $Coord_{patch}$ **in** $List_{coord}$ **do**8:     $I_{patch}\leftarrow \mathrm{Tailor}\left(I_{pre},Coord_{patch},Shape_{patch}\right)$;9:     $Cls_{ABox},Reg_{ABox}\leftarrow \mathrm{Detector}\left(I_{patch},Set_{ABox},Coord_{patch}\right)$;10:    $Candis\leftarrow Cls_{Abox}\left[Cls_{Abox}\geq C_{th}\right],Reg_{Abox}\left[Cls_{Abox}\geq C_{th}\right]$;11:    Stores candidates of the patch Candis to the set $Set_{candi}$;12:**end for**13:$Set_{candi}\leftarrow \mathrm{LMS}\left(Set_{candi},I_{mask}\right)$, $Set_{candi}\leftarrow {\mathrm{NMS}}_{\mathrm{IOU}}\left(Set_{candi}\right)$;14:**for** Candi **in** $Set_{candi}$ **do**15:    $I_{candi}\leftarrow \mathrm{Tailor}\left(I_{pre},Coord_{candi},Shape_{candi}\right)$;16:    $Candi_{score}\leftarrow \mathrm{Estimator}\left(I_{candi}\right)$;17:     **if** $Candi_{score}\geq C_{s}$ **then**18:         Stores this candidate Candi in $List_{n}$;19:      **end if**20:**end for**21:**return** $List_{n}$.

#### 3.2.1. Preprocessing

As shown in Figure 4a, the scan contains some interference regions, such as the body trunk, imaging equipment, and air areas. In fact, nodule detection only focuses on the lung region. Thus, a quick way to proceed is to extract the lung region and strip irrelevant regions for the LDCT scans [42]. In our CAD system, pre-image [Figure 4h] and lung-mask [Figure 4e] are obtained by the following preprocessing method.

**Pulmonary Parenchyma Segmentation.** This procedure can be split into four steps. (1) The spacing of the raw images is re-sampled to [1.0,1.0,1.0] using bilinear interpolation. (2) A threshold filter with a Hounsfield Unit (HU) of −600 is used to obtain a binarized image as shown in Figure 4b. (3) The air region is segmented by region growing, which selects fixed vertexes of the binarized image as initial seed points. Then, a body mask [Figure 4c] is obtained by sequentially using a non-logical operation and extracting a max-connected domain. (4) The binarized image subtracted from the body mask is a coarse full lung mask. Then, a full clean lung mask [Figure 4d] is obtained using a sphere structuring element with a radius of 6 to dilate the coarse lung mask.

**Pre-Image and Lung-Mask Extraction.** Subsequently, the following steps are adopted to extract the final results. (1) We calculate a min bounding-box of the max-connected domain in Figure 4d. (2) A lung-mask and a pre-image are extracted from the clean mask and raw image respectively, by using the bounding-box as an index range. (3) We convert the HU range of pre-image from [−1200,600] to [0,255] through a linear transformation, as shown in Figure 4g. (4) The final pre-image is obtained by filling Figure 4g with a gray-scale of 170 treating the lung-mask as an index map.

#### 3.2.2. Two-Stage Nodule Detection

In the first stage, the MSFD-Net predicts the classification probability and four regression offsets of each anchor-box that is predefined in a 3D patch, where the 3D patch is extracted from pre-image by a sliding window with a stride of 210, and reshaped as 1×240×240×240. Subsequently, all anchor-boxes in which the probability is greater than a candidate threshold of 0.75 are considered rough nodule candidates. The predicted coordinate and diameter of the candidate [z,y,x,d] are obtained using the predicted offsets to refine their own predefined coordinates and diameter. Finally, a candidate suppression algorithm removes many nodule candidates by non-maximum suppression based on intersection over union (NMS-IOU) and lung mask suppression (LMS). The remaining nodule candidates are regarded as the detection results of one-stage MSFD-Net.

In the second stage, for each candidate, a 3D candidate patch is extracted from the pre-image according to predicted coordinates and is reshaped as 1×32×32×32. Moreover, the CS-Net estimates the score of each candidate patch. Finally, we discard candidates whose score is lower than the predefined threshold of 0.05 to realize a false positive reduction.

### 3.3. Nodule Candidate Detection

**Figure 5:**
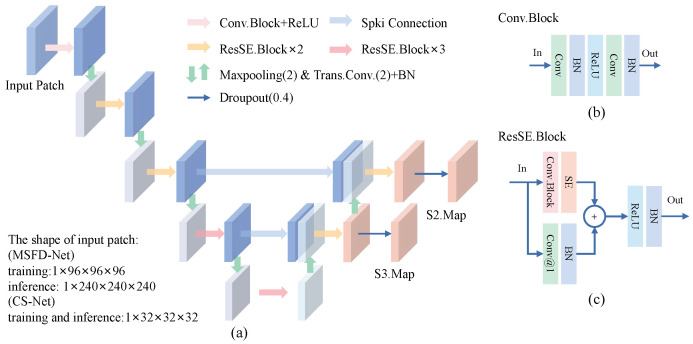
The architecture of the proposed multi-scale feature extractor (MSFE). (**a**) The MSFE extracts the multi-scale features S2.Map and S3.Map from the input patch. (**b**) Structure of the basic module Conv.Block. (**c**) Structure of the basic module ResSE.Block. Conv@1 means a 3D convolution with a kernel size of 1×1×1. In our modules, note that the kernel size of convolutions is 3×3×3, and the stride and padding are 1 unless otherwise specified.

**Multi-Scale Feature Extractor.** As shown in Figure 5a, we design the architecture of a 3D multi-scale feature extractor by making modifications to the feature pyramid network [35]. Moreover, we build Conv.Block [Figure 5b] and ResSE.Block [Figure 5c] to construct the proposed MSFD. A SE-Block attention [39] is adopted in ResSE.Block. It can inspire the network to adjust the weights of each channel through inter-dependence between channels obtained by the spatial encoding of features.

The encoding path of MSFE is composed of five convolutional encoding layers to learn the feature representation of nodule candidates. There is a max-pooling module to down-sample the feature between each encoding layer. The 1st layer consists of one Conv.Block and one ReLU activation. Layers from 2 to 5 are built to stack ResSE.Blocks repeatedly. The decoding path of MSFE contains two cascaded feature decoding layers. Each decoding layer is composed of one transpose convolution and two ResSE.Blocks. During the training phase, we introduce dropout regularization to prevent over-fitting of the network.

Let the shape of the input 3D patch be 1×d×h×w, where d,h and *w* mean the depth, height, and width of the patch, respectively. The features of nodule candidates S2.Map with shape of $c\times \frac{d}{4}\times \frac{h}{4}\times \frac{w}{4}$ and S3.Map with shape of $c\times \frac{d}{8}\times \frac{h}{8}\times \frac{w}{8}$ are extracted by the MSFE forward.

In this work, we implement two MSEFs with different amounts of parameters. They are used to construct an MSFD-Net to generate nodule candidates and CS-Net to evaluate the score of candidates, respectively, where the number of MSFE parameters used by CS-Net is far less than that used by MSFD-Net.

**Figure 6:**
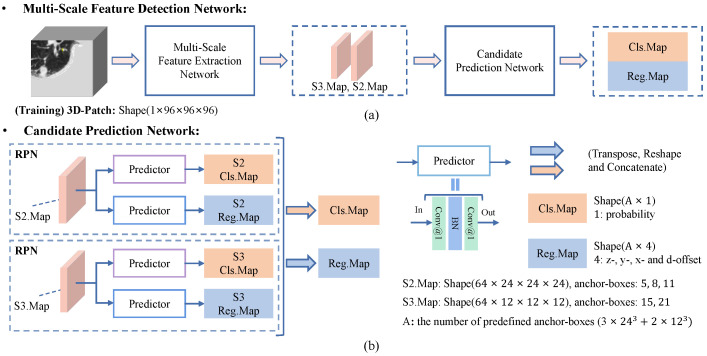
Flowchart of the proposed multi-scale feature detection network (MSFD-Net). (**a**) The MSFD-Net consists of a multi-scale feature extractor and candidate prediction network (CPN). (**b**) The CPN predicts one classification map (Cls.Map) and four regression offset maps (Reg.Map) of candidates at two scales, respectively.

**Multi-Scale Feature Detection Network.** The framework of the proposed multi-scale feature detection network is shown in Figure 6. In MSFD-Net, an MSFE first extracts feature vectors of each predefined anchor-point. Then, a candidate prediction network (CPN) [Figure 6b] predicts the classification map Cls.Map and regression map Reg.Map for all anchor-boxes. Different from single-scale nodule detection methods [9,12,16,17,23,24], the CPN consists of two RPNs to achieve multi-scale prediction task. Each RPN [22,43] contains two predictors constructed by two convolutions and one batch-norm to predict results at a single scale.

In the training phase, the number of predefined anchor-points is $24^{3}$ in S2.Map, and $12^{3}$ in S3.Map, when the space shape of input 3D patch is 96×96×96. An anchor-box preset on the anchor-point is defined by four parameters: (*z*, *y*, *x*, *d*), where the first three values (*z*, *y*, *x*) mean the predefined location of the anchor-point and anchor-box in the patch, and the *d* means the predefined diameter of the anchor-box. In this work, we predefined three anchor-boxes with diameters of 5, 8, and 11 on each anchor-point in S2.Map, and two anchor-boxes with diameters of 15 and 21 on each anchor-point in S3.Map. The small nodules are detected using fine-resolution S2.Map, and large nodules are detected using norm resolution S3.Map. Its aim is to adapt the variation in nodule size and imbalance in the number of nodules of different sizes, in the LUNA16 dataset.

As shown in Figure 6b, two RPNs predict the classification and regression map of anchor-boxes from the feature maps extracted by MSFE at their respective scale. Then the classification and regression maps of each scale are integrated into Cls.Map (A×1) and Reg.Map (A×4) by tensor operations, respectively, where *A* is the number of anchor-boxes and equal $3\times 24^{3}+2\times 12^{3}$. Finally, the number of parameters is about 3.30 million in MSFD-Net.

**Label Assignment Strategy.** According to a nodule label $G(g_{z},g_{y},g_{x},g_{d})$, we randomly crop a training patch from its pre-image and reshape it as 1×96×96×96. We design an online random data augmentation strategy that consists of random flipping, zooming, and transposing, to randomize and augment the data as much as possible. It can make the best use of limited labeled data.

A label match strategy is designed to distribute each anchor-box to different sample sets. The strategy can be split into three steps as follows. (1) We calculate Intersection over Union (IoU) between all anchor-boxes and each label in an extracted patch. (2) For each label, we randomly choose one of anchor-boxes that satisfies IoU≥0.5, and consider it as a positive sample of the label; if any anchor-box does not satisfy IoU≥0.5, then the anchor-box of maxIoU chooses. Afterward, a positive-box set ($Set_{P}$) is defined by all positive samples. (3) We randomly sample 4000 anchor-boxes that satisfy IoU≤0.02 with any nodule label, and conceived them as negative samples. They also define a negative-box set ($Set_{N}$). The remaining anchor-boxes that are not selected select define an ignored-box set ($Set_{I}$).

There is a label $G(g_{z},g_{y},g_{x},g_{d})$ that matches a positive sample $B(b_{z},b_{y},b_{x},b_{d})$ in $Set_{P}$. The regression offset label $R(r_{z},r_{y},r_{x},r_{d})$ of the label is obtained as follows:

(1) $$r_{i}=\frac{g_{i}-b_{i}}{b_{d}},\left(i=z,y,x\right),r_{d}=\ln\left(\frac{g_{d}}{b_{d}}\right).$$

**Loss Function.** In this work, we use Focal Loss [44] and Smooth L1-Norm Loss to supervise the learning of MSFD-Net. First, we predict the probabilities ($p^{*}$) of all anchor-boxes are obtained by inputting Cls.Map into a sigmoid activation. In addition, the category label p=1 means that the anchor-box is positive, and the negative for the label p=0. The Focal Loss $L_{f}\left(p^{*},p\right)$ is defined as follows:

(2) $$L_{f}\left(p^{*},p\right)=\left\{\begin{array}{cc} \alpha \times {\left(1-p^{*}\right)}^{\gamma }\times \log\left(p^{*}\right), & p=1\\ \left(1-\alpha \right)\times {\left(p^{*}\right)}^{\gamma }\times \log\left(1-p^{*}\right), & p=0 \end{array}\right.$$

where the balance factor α is set as 0.5, and the focusing factor γ is set as 2.0. Furthermore, the category loss $L_{\mathrm{cls}}$ based on a batch-patch can be obtained by the following:

(3) $$L_{cls}=\frac{1}{N}\times \sum_{i=1}^{N}L_{f}\left(p_{i}^{*},p_{i}\right),$$

where *N* means the number of samples sampled by online hard negative mining described (OHNM) [45] in the following section. Second, Smooth L1-Norm Loss $L_{s}\left(r^{*},r\right)$ is obtained by the following:

(4) $$L_{s}=\left\{\begin{array}{cc} {\left(r^{*}-r\right)}^{2}, & |r^{*}-r|<1\\ |r^{*}-r|, & |r^{*}-r|\geq 1 \end{array}\right.$$

where the tokens $r^{*}$ and *r* are denoted as the prediction and label of regression offsets, separately. The regression loss $L_{\mathrm{reg}}$ based on a batch-patch is expressed by the following:

(5) $$L_{reg}=\frac{1}{K}\times \sum_{i=1}^{K}\sum_{j}L_{s}\left(r_{ij}^{*},r_{ij}\right),j=\left(z,y,x,d\right),$$

where *K* means the number of positives in the batch patch.

Finally, the total loss based on a batch patch is calculated by the following:

(6) $$L_{total}=L_{cls}+L_{reg}.$$

**Online Hard Negative Mining.** In a batch patch, an obvious class-imbalance problem exists; In other words, the number of samples in all $Set_{P}$ is far less than that in all $Set_{N}$. The effective online hard negative mining strategy [45] is used to process this problem. Firstly, the negative samples are sorted by descending order of their Cls.Map. Second, the top-*k* negative samples are selected and treated as the hard-to-distinguish negative samples, where *k* is β times the number of the positive sample (*K*). Finally, the *N* samples are fed into $L_{cls}$ to calculate the classifying loss. By adjusting the value of β, the ratio of losses between positive and negative samples can be controlled, thereby alleviating the imbalance problem.

**Candidate Suppression Algorithm.** After MSFD-Net detection, there are many candidates that are adjacent to or outside the lung parenchyma. To provide more accurate candidates for clinicians, a candidate suppression algorithm is constructed.

First, we adopt non-maximum suppression based on IoU to overcome the adjacent detection. (1) A ranking table in ascending order is obtained by sorting the candidate’s probabilities. (2) The 1st candidate is selected and treated as a reference candidate in the ranking table. Then, we discard many candidates whose IoU between the reference candidate is greater than 0.001 from the ranking table. (3) The previous step is repeated until all candidates in the ranking table are selected or discarded.

Second, we propose a lung mask suppression (LMS) algorithm to remove candidates outside the lung parenchyma. (1) The lung-mask obtained from the preprocessing stage is considered a density field; the density value of the foreground is 1, and the background is 0. (2) Each selected candidate is regarded as a 3D sphere; then, the mean density $C_{m}$ of the candidate is calculated by the following:

(7) $$C_{m}=\frac{1}{V_{sphere}}\times \sum_{\vec{p}}L_{mask}\left(\vec{p}\right),$$

where $V_{sphere}$ means the volume of a sphere. $L_{mask}\left(\vec{p}\right)$ means the density value on point $\vec{p}=\left(p_{z},p_{y},p_{x}\right)$, and any point satisfies the following inequalities:

(8) $$\frac{4}{{q_{d}^{*}}^{2}}\times {\left(p_{z}-q_{z}^{*}\right)}^{2}\times {\left(p_{y}-q_{y}^{*}\right)}^{2}\times {\left(p_{x}-q_{x}^{*}\right)}^{2}\leq 1,$$

where $\left(q_{z}^{*},q_{y}^{*},q_{x}^{*}\right)$ means the predicted position of candidates. Note that the range of $C_{m}$ is from 0 to 1. (3) We preserve candidates with $C_{m}$ more than 0.8 as detection results of one-stage MSFD-Net.

### 3.4. False Positive Reduction

In the lung, many nodule-like tissues existed, such as blood vessels, airway walls, and lung aberrations [31]. Thus the nodule detection algorithms often considered them as nodule candidates (i.e., false positives) [25,26]. This issue can reduce the performance of the detection algorithm to some extent. Therefore, a strategy to reduce false positives needs to be implemented to ensure performance.

**Figure 7:**
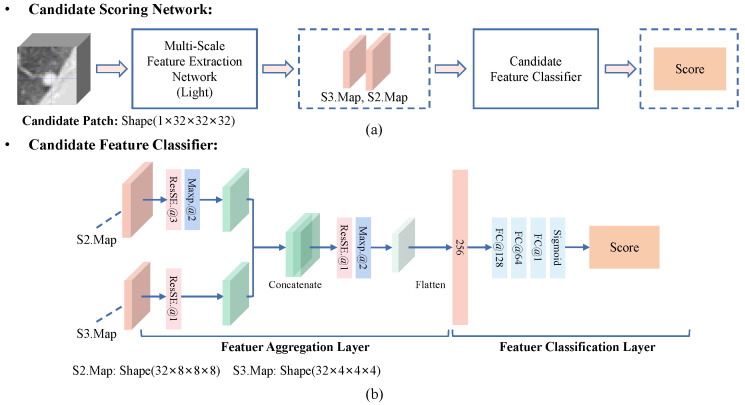
Flowchart of the proposed candidate scoring network (CS-Net). (**a**) The MSFD-Net consists of a lightweight multi-scale feature extractor and a candidate feature classifier. (**b**) The candidate feature classifier estimates the score of a candidate by integrating the multi-scale features of the candidate.

**Candidate Scoring Network.** As shown in Figure 7, we propose a false positive reduction network called a candidate scoring network. It achieves binary classification of candidates into nodules and non-nodules by evaluating the scores of candidate patches. In CS-Net, we use a lightweight MSFE to extract multi-scale features of candidate patches and design a candidate feature classifier to evaluate the score of the candidates. As shown in Figure 7b, the features of S2.Map with the shape of 32×8×8×8 and S3.Map with the shape of 32×4×4×4 are integrated into a feature vector with a length of 256 by a feature aggregation layer. Then, the feature vector is fed into a feature classification layer to estimate the score of the candidate patch with the shape of 1×32×32×32. Finally, the number of parameters is about 1.52 million in CS-Net.

**Offline Data Augmentation.** We plan an offline data augmentation (Off-Aug). It aims to balance the ratio between positives and negatives in the candidate dataset provided by the LUNA16 dataset. For each positive candidate in the dataset, the augmentation is described as follows. (1) The strategy loads the coordinates of a candidate in a pre-image, and the pre-image is zoomed by a scale factor that is randomly selected from [0.8, 1.25]. (2) The coordinate of each axis is moved for a short distance that is randomly selected from the range of [−4,4]. (3) A 3D candidate patch is cropped from the pre-image and reshaped as 1×32×32×32, by considering the moving coordinates as the cropping center. (4) The patch is randomly flipped and transposed to obtain the augmented patch. For each positive candidate, the procedure is executed 40 times. For negative candidates in the dataset, only a candidate patch is cropped according to its coordinates. In the end, all extracted candidate patches are stored on a disk.

### 3.5. Implementation Details

We mainly use algorithm libraries SimpleITK [46] and Pytorch [47] to implement our proposed nodule CAD system. The training and evaluating stage was performed on GTX 2080Ti with 12GB memory.

**Details about Training MSFD-Net.** To alleviate the problem of imbalance in labeled nodules in LUNA16, as shown in Figure 2. For each labeled nodule, its frequency loaded repeatedly $l_{f}$ in the training set is determined by the expression of $l_{f}=1+\left\lfloor d/10\right\rfloor \times 3$, where *d* means its diameter. We use Adam optimizer with default parameters to update the weights of the network. The batch size is 10, and 300 for training epochs. The initial learning rate $l_{\alpha }$ is 0.001, and this is then adjusted as the initial of 0.1, 0.05, and 0.01 after 60, 120, and 180 epochs, respectively. Moreover, based on the batch patch, the ratio of the number of negatives and positives β is set to 10 for the OHNM.

**Details about Training CS-Net.** In the training stage, the candidate patches obtained by Off-Aug are loaded to train the proposed CS-Net with a batch size of 100. The training epoch is set to 12. The training dataset is shuffled, and only 70% negatives are randomly sampled and regarded as training negative samples before each training epoch begins. This aims to further control the problem of class imbalance and alleviate data dependency. Moreover, the β is set to 5 for the OHNM. The settings of the optimizer and initial learning rate are consistent with the MSFD-Net.

## 4. Results

In our experiments, we chiefly verify the FROC performance of nodule detection methods based on MSFD-Net. Specifically, the same MSFD-Net is first used as the basis. Then, the candidate suppression algorithm (NMS-IoU and LMS) and CS-Net are introduced stage by stage. Finally, baseline, one-stage MSFD-Net (OS-MSFD), and two-stage MSFD-Net (i.e., our proposed TSND as shown in Figure 3) are obtained separately.

### 4.1. Comparison Based on FROC

The FROC metrics of the above methods are listed in Table 2. The baseline achieves a better CPM value of 87.31%, which is slightly less than that achieved by OS-MSFD (88.17%) and TSND (90.59%). The highest sensitivity of 97.04% at 8.0 FPs/Scan is reached by OS-MSFD. In addition, the sensitivity of TSND excels OS-MSFD at the first five FPs/Scan points, but it is slightly inferior at the two last points.

These results indicate three conclusions. First, the proposed MSFD-Net can generate candidates with high confidence from LDCT scans. This is due to the fact that MSFE extracts multi-scale features, thereby providing features with different spatial resolutions. The CPN can predict small-scale candidates on the fine features and large-scale on the coarse feature, which adapts well to the diameter variation of nodules. Second, the designed candidate suppression algorithm reliably removes some candidates that are detected repeatedly or generated at unexpected locations. Third, the CS-Net integrated into TSND can efficiently eliminate a lot of false positives. It is realized to improve the mean sensitivity, whereas false positives have remained at a low level.

### 4.2. Comparison Based on FROC and Execution Time

We use CAD Performance [25] to evaluate candidates generated by our methods. Table 3 shows the evaluation results of candidates. The columns separately mean the FPs/Scan, sensitivity, and precision of nodule detection. In addition, The average execution times per scan of the three methods also are listed in the last column of Table 3.

First, at three evaluation items, the baseline has reached 13.51, 97.26%, and 12.29%. Second, the LMS algorithm is adopted to remove invalid candidates. The FPs/Scan of OS-MSFD drops to 8.33, whereas the sensitivity of 97.18% is maintained at the same plane, and the precision increases to 18.51% in comparison with the baseline. Third, Table 3 illustrates that the TSND obtains the FPs/Scan of 2.53, and the precision of TSND increases to 42.44%. These results benefit from the false positive reduction provided by the proposed CS-Net. In terms of average execution time, the proposed TSND average execution time is 3.0655s per scan, which is 1.04% and 0.77% slower than Baseline and OS-MSFD, respectively.

### 4.3. The Effectiveness of CS-Net

**Figure 8:**
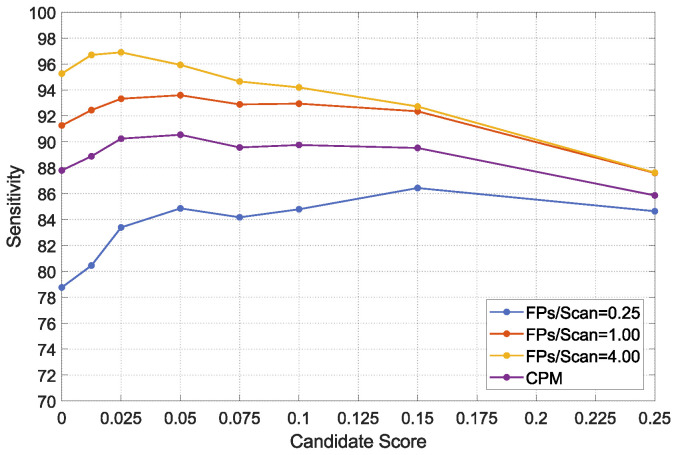
FROC values of the TSND using the CS-Net adopted different candidate scores on the validation set.

In this section, we design an experiment to explain the effectiveness of the proposed CS-Net removes false positives. First, we select the three subsets from 10 subsets of the LUNA16 dataset as a validation set. Subsequently, we preset eight candidate scores, i.e., 0, 0.0125, 0.025, 0.05, 0.75, 0.1, 0.15, and 0.25, respectively. The score of 0 means that the CS-Net is not enabled. In the validation set, the FROC values of TSNDs with different candidate scores are listed in Table 4 and drawn in Figure 8.

As shown in Table 4, the detection performance of TSND is enhanced, when the first few lower scores are adopted. Specifically, in the case of sacrificing only a small amount of detection sensitivity, FROC and CPM values increase obviously, whereas the FPs/Scan are greatly reduced. For example, the FROC value at 1.0 FPs/Scan increases from 91.26% to 93.59%, and the FPs/Scan decreases from 11.48 to 3.02. However, the sensitivity decreases from 97.20% to 95.97%, when the score of 0.05 is adopted. Moreover, when the scores rise to a certain threshold, with the increasing score, FROC values decline, because the CS-Net eliminates too many true positives. In summary, the score has a suitable balance point that can not only effectively suppress false positives, but also improve the detection performance.

### 4.4. Visualization

**Figure 9:**
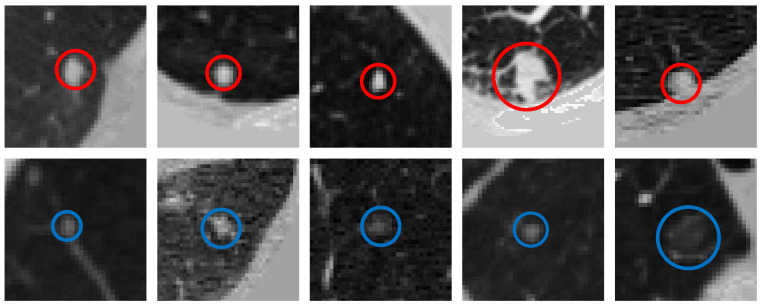
Visualization of labeled nodules. The first row shows the nodules correctly predicted by our MSFD-Net (red solid line circles). And the last row shows the missed nodules, of which a solid blue line circle illustrates the ground truth.

As described in the above experiments, our proposed method obtains the first-class sensitivity of nodule detection. However, MSFD-Net also misses some difficult nodules to classify, such as small nodules that adhere to blood vessels, those that are interfered with by noise and those with obscure image features. Figure 9 shows some predicted or missed nodules.

**Figure 10:**
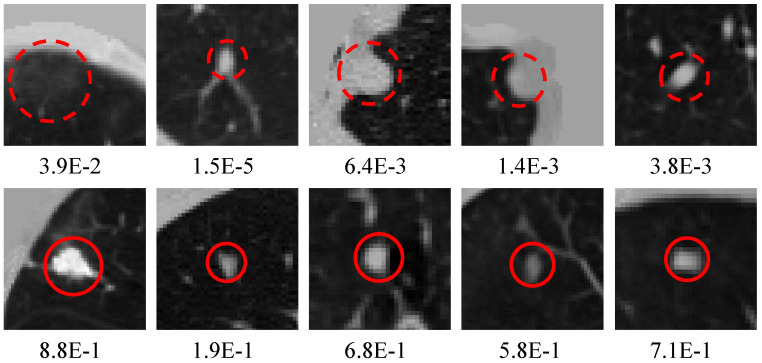
Visualization of candidates generated by the MSFD-Net. The red dotted circles indicate false positives and the red solid line circles are for true positives. Besides, the numeric note under each slice means the score estimated by CS-Net.

Figure 10 shows the candidates generated by the MSFD-Net and their scores evaluated by the CS-Net. Many suspected objects are regarded as nodule candidates to guarantee that the missed nodules are as few as possible, in the detection stage. Hence the MSFD-Net produces many false positive candidates, examples of which are inflammation, aberrations distortion, blood vessels, chest walls, and others.

**Figure 11:**
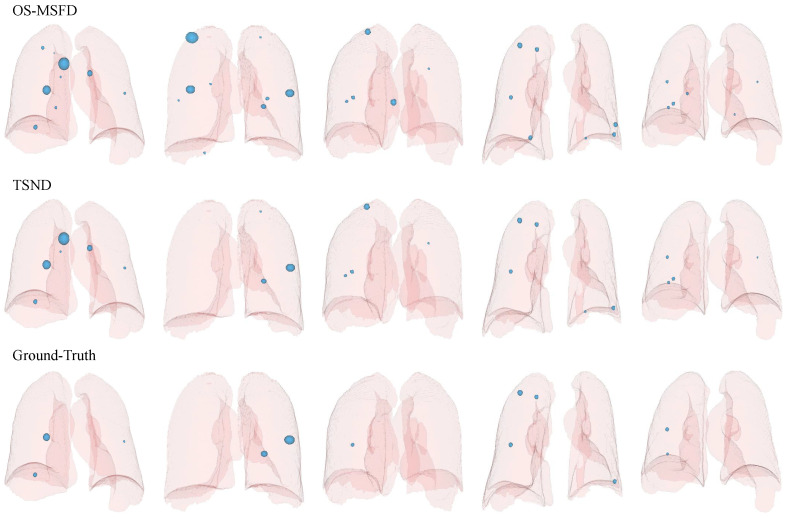
Visualization of candidates (ground-truths) in the 3D lung mask label, where 3D light blue spheres represent predicted candidates (ground-truths). The first two rows indicate the candidates detected by OS-MSFD and TSND, respectively, and the last row is for ground truth supported by the LUNA16 official.

We plot candidate nodules detected in some scans in their respective 3D lung mask labels to enable visualization of OS-MSFD and TSND detection results. Nodule labels for these scans also are drawn to facilitate comparison. The results of the visualization are shown in Figure 11. The visualization results indicate that the nodules detected by the TSND are close to the ground truths.

## 5. Discussion

### 5.1. Effect of Nodule Size on Detection

The size of pulmonary nodules plays a pivotal role in differentiating benign and malignant nodules The size of pulmonary nodules plays a pivotal role in diagnosing benign and malignant nodules for clinicians [16]. In this section, we analyze the detection results of nodules of different sizes. First, labeled nodules are classified into three categories based on their diameter: small [3,10)mm, medium [10,20)mm, and large [20,30)mm. Second, we collected and collated detection results of three proposed methods, which are listed in Table 5.

**Figure 12:**
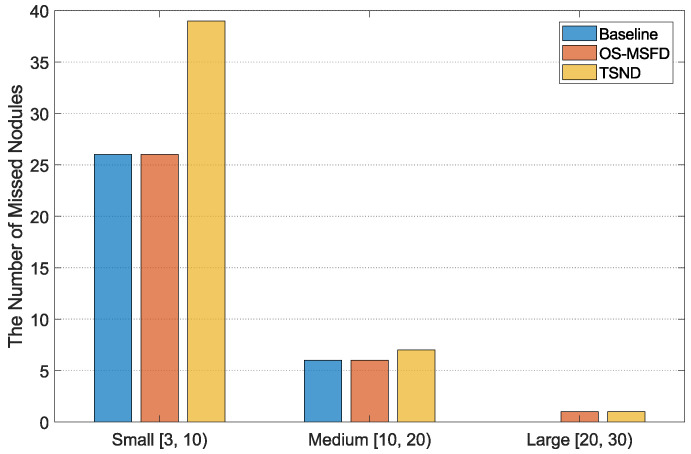
The distribution of missed nodules of different sizes for three methods.

Figure 12 counts the distribution of missed nodules of different sizes for three methods. Regarding the number of missed nodules detected by MSFD-Net, small nodules are the most numerous, followed by medium ones, and large ones are the least numerous. Meanwhile, the small nodules are the most incorrectly classified by CS-Net compared with the other two types of nodules.

As illustrated in Table 5, the proposed MSFD-Net has recalled the nodules of different sizes. The multi-scale feature extraction and prediction strategy are adopted. It can adapt well to the variation of nodules in diameter. Moreover, the proposed two-stage method obtained the best detection precision for nodules of different sizes. Cascading the false positive reduction network after the detection network is an effective strategy.

### 5.2. Statistical Significance Analysis of Differences in Detection Precision

We used paired t-tests [48] to indicate whether the difference in detection precision among TSND, Baseline, and OS-MSFD are statistically significant. The variable of $\tau_{t}^{AB}$ is defined as follows:

(9) $$\tau_{t}^{AB}=\left|\frac{\sqrt{k}\times \mu }{\sigma }\right|∼t\left(k-1\right),$$

where the AB means a pair of nodule detectors, the k=10 means the number of subsets in the LUNA16 dataset, and the t(k−1) is a *t*-distribution with k−1 degree of freedom. The μ and σ are illustrated as follows:

(10) $$\delta_{i}=P_{i}^{A}-P_{i}^{B},\;\mu =\frac{1}{k}\sum_{i=1}^{k}\delta_{i},\;\sigma =\sqrt{\frac{1}{k-1}\sum_{i=1}^{k}{\left(\delta_{i}-\mu \right)}^{2}},$$

where, the $P_{i}^{A}$ and $P_{i}^{B}$ respectively represent the precision of detectors *A* and *B* on *i*-th subset.

Under the significance level of α=0.05, the critical value of $t_{\frac{\alpha }{2},k-1}$ equals to 2.262. If the $\tau_{t}^{AB}\geq t_{\frac{\alpha }{2},k-1}$ is valid, than the difference in precision detectors *A* and *B* is statistically significant. According to Equations (9) and (10), $\tau_{t}^{AB}$ between TSND and Baseline equals to 16.011, $\tau_{t}^{AB}$ between OS-MSFD and Baseline equals to 2.883, and $\tau_{t}^{AB}$ between TSND and OS-MSFD equals to 11.011. The results support a statistically significant difference in detection precision among TSND, baseline, and OS-MSFD.

### 5.3. Comparison with Other Detection Methods

This section compares several state-of-the-art nodule detection methods with the proposed TSND on the LUNA16 dataset. The selected solutions can be divided into one-stage methods (N-Net [12], DeepSEED [22], CPM-Net [27], and SCPM-Net [23]), and the two-stage methods (FPN+CNN [19], FRCN+3DCNN [18], Nodule-Net [21] and SA-Net [16]). The results of selected methods are provided in the official works rather than re-implemented.

**Figure 13:**
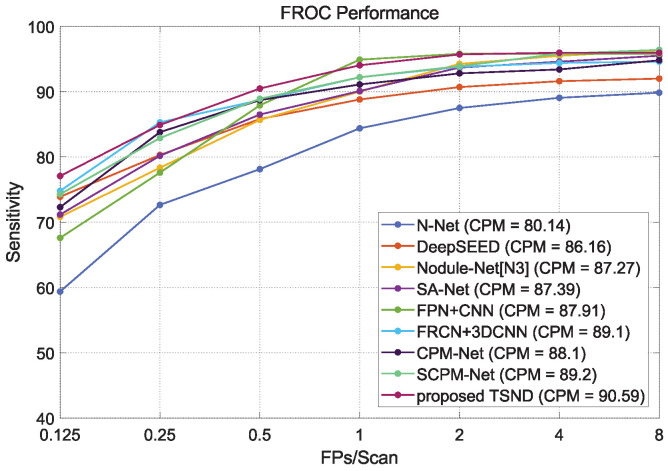
The comparison of FROC curves between other state-of-the-art methods and our proposed two-stage nodule detection (TSND) method.

FROC curves and CPM values of compared methods and proposed TSND are listed in Table 6 at seven FPs/Scan points. Furthermore, FROC curves are illustrated in Figure 13. Our TSND achieves an excellent level compared with other state-of-the-art methods on the metric of CPM value. The TSND achieves a CPM value of 90.59%.

In addition, our TSND preserves higher detection sensitivity at low FPs/Scan points compared with most other methods. For example, the TSND achieves 77.08% sensitivity at 0.125, 90.48% sensitivity at 0.5, and 94.04% sensitivity at 1.0 for different FPs/Scan points, and these are significantly higher than most other methods. It can be ascribed to the second stage CS-Net which removes many false positive candidates generated by the first stage MSFD-Net.

Moreover, the one-stage methods have better sensitivity under the highest FPs/Scan points. Specifically, the best sensitivity of 96.40% at 8.0 FPs/Scan is achieved by the SCPM-Net. This result can be attributed to the listed two-stage methods of integrating an FPR strategy that removes false positive candidates while a few nodules are also inevitably considered. For our TSND, although the sensitivity decreases slightly at the last FPs/Scan point, the overall average sensitivity increases, and the number of false positives drops even more. As a result, the TSND can provide more precise candidates for a clinician’s nodule diagnosis.

## 6. Conclusions

Overall, the experimental results show that our proposed TSND is an efficient and usable solution for lung nodule detection. We hope that our proposed solution can provide a valuable reference for the clinical application of deep learning for pulmonary nodule diagnosis and lung cancer screening. In the future, we will conduct research on nodule segmentation and classification and finally develop a complete end-to-end nodule-aided diagnosis system.

## Figures and Tables

**Table 1:** Summary of the Related Works. False Positive Reduction (FPR). The token of ‘w’ (‘w/o’) means the listed method with (without) the FPR strategy.

Authors	Year	Dataset	Method	FPR
Han et al. [9]	2022	LUNA16 and LNPE1000	UNet+RPN, U-shaped Net (UNet) [40] Region-based Proposal Network (RPN) [36]	w/o
Liao et al. [12]	2019	LUNA16 and DSB	N-Net, where the character N means the nodule	w/o
Zhu et al. [20]	2018	LUNA16 and LIDC-IDRI	DeepLung	w/o
Song et al. [27]	2020	LUNA16	Center-points Matching Network (CPM-Net)	w/o
Luo et al. [23]	2022	LUNA16	Sphere Center-points Matching Network (SCMP-Net)	w/o
Huang et al. [24]	2022	LUNA16	You Only Look Once version 3 (YOLOv3) [37]	w/o
Li et al. [22]	2020	LUNA16 and LIDC-IDRI	Deep Squeeze-and-Excitation Encoder-Decoder (DeepSEED)	w/o
Dou et al. [17]	2017	LUNA16	Convnets	w
Tang et al. [21]	2019	LUNA16	Nodule-Net	w
Mei et al. [16]	2021	LUNA16	Slice-Aware Network (SA-Net)	w
Ding et al. [18]	2017	LUNA16	Faster Region-based CNN (FRCN) [36]	w
Ozdemir et al. [28]	2019	LUNA16	V-Net+3DCNN, Volumetric Convolutional Network (V-Net) [41]	w
Wang et al. [19]	2018	LUNA16	FPN+CNN, Feature Pyramid Network (FPN) [35]	w

**Table 2:** Comparison of the FROC metrics with three methods based on the proposed MSFD-Net using the 10-fold cross-validation on the LUNA16 dataset. The sensitivities (%) are shown in the following table, at seven FPs/scan points 0.125, 0.25, 0.5, 1, 2, 4, and 8 on the FROC curve of different methods. Besides, the CPM means the average sensitivity at the seven points described above.

Method	FROC							
	0.125	0.25	0.5	1.0	2.0	4.0	8.0	CPM
Baseline	69.68	79.38	85.54	90.55	93.53	95.82	96.71	87.31
OS-MSFD	71.87	80.50	86.37	91.08	94.16	96.13	97.04	88.17
TSND	77.08	84.90	90.48	94.04	95.70	95.97	95.97	90.59

**Table 3:** Comparison of candidate detection results of three proposed methods on LUNA16 dataset using LUNA16 CAD Performance and their average execution times (unit:s) per scan (ET/Scan).

Method	FPs/Scan	Sensitivity	Precision	ET/Scan
Baseline	13.51	97.26	12.29	3.0334
OS-MSFD	8.33	97.18	18.51	3.0422
TSND	2.53	95.98	42.44	3.0655

**Table 4:** Comparison of the FROC values and LUNA16 CAD Analysis of the TSND with the CS-Net using different candidate scores on the validation set.

Score	FROC				FPs/Scan	Candis/Scan	Sensitivity
	0.25	1.0	4.0	CPM			
0.0000	78.76	91.26	95.26	87.79	11.48	17.04	97.20
0.0125	80.45	92.44	96.70	88.88	5.58	10.64	96.89
0.0250	83.39	93.32	96.90	90.24	4.13	8.97	96.89
0.0500	84.86	93.59	95.93	90.54	3.02	7.53	95.97
0.0750	84.17	92.88	94.65	89.56	2.67	7.06	94.66
0.1000	84.79	92.94	94.19	89.75	2.19	6.45	94.14
0.1500	86.43	92.35	92.72	89.52	1.67	5.59	92.79
0.2500	84.64	87.59	87.62	85.86	1.24	4.60	87.60

**Table 5:** Comparison of the detection sensitivity(%) and precision(%) of three proposed methods: Baseline, OS-MSFD, and TSND for nodules of different sizes.

Method	Sensitivity				Precision		
	Small	Medium	Large		Small	Medium	Large
Baseline	97.09	97.39	100.0		15.19	7.23	10.45
OS-MSFD	97.09	97.39	97.92		17.49	23.30	20.40
TSND	95.63	96.99	97.92		40.65	49.40	48.86

**Table 6:** Comparison of the FROC metrics between other state-of-the-art methods and our proposed two-stage nodule detection (TSND) using the 10-fold cross-validation on the LUNA16. (FPR: false positive reduction).

Method	FROC								FPR	Strategy
	0.125	0.25	0.5	1.0	2.0	4.0	8.0	CPM		
N-Net [12]	59.38	72.66	78.13	84.38	87.50	89.06	89.84	80.14	w/o	Anchor-Box
DeepSEED [22]	73.90	80.30	85.80	88.80	90.70	91.60	92.00	86.16	w/o	Anchor-Box
Nodule-Net[N3] [21]	70.82	78.34	85.68	90.01	94.25	95.49	96.29	87.27	w	Anchor-Box
SA-Net [16]	71.17	80.18	86.49	90.09	93.69	94.59	95.50	87.39	w	Anchor-Box
FPN+CNN [19]	67.60	77.60	87.90	94.90	95.80	95.80	95.80	87.91	w	Anchor-Box
CPM-Net [27]	72.30	83.80	88.70	91.10	92.80	93.40	94.80	88.10	w/o	Anchor-Free
FRCN+3DCNN [18]	74.80	85.30	88.70	92.20	93.80	94.40	94.60	89.10	w	Anchor-Box
SCPM-Net [23]	74.30	82.90	88.90	92.20	93.90	95.80	96.40	89.20	w/o	Anchor-Free
proposed TSND	77.08	84.90	90.48	94.04	95.70	95.97	95.97	90.59	w	Anchor-Box

## Data Availability

LUNA16 dataset can be found at https://luna16.grand-challenge.org/Download/, accessed on 24 March 2016.
